# Unlocking Demography: Developing an eDNA‐Based Toolkit to Measure Sex Ratios From Populations

**DOI:** 10.1111/1755-0998.70089

**Published:** 2025-12-18

**Authors:** Emilie A. Didaskalou, James France, Milena Cvijanović, Krijn B. Trimbos, Tijana Vučić, Maja Ajduković, Ana Ivanović, Ben Wielstra, Peter M. van Bodegom, Kathryn A. Stewart

**Affiliations:** ^1^ Institute of Environmental Sciences Leiden University Leiden the Netherlands; ^2^ Institute of Biology Leiden University Leiden the Netherlands; ^3^ Naturalis Biodiversity Centre Leiden the Netherlands; ^4^ Institute for Biological Research “Siniša Stanković”—National Institute of the Republic of Serbia University of Belgrade Belgrade Serbia; ^5^ Faculty of Biology University of Belgrade Belgrade Serbia

**Keywords:** amphibian, biodiversity monitoring, demography, environmental DNA, population dynamics

## Abstract

Demographic information, such as sex ratios, is essential for understanding population dynamics and informing conservation strategies. Yet obtaining sex ratios in natural populations can be challenging due to logistical, ethical and legal constraints. Environmental DNA (eDNA) has revolutionised non‐invasive biodiversity monitoring, but its potential for assessing demographic parameters remains largely unexplored. Here we present an eDNA‐based method to monitor sex ratios of populations by quantifying sex‐specific SNP alleles. Using RADseq data from Balkan crested newts ( *Triturus ivanbureschi* ), we identified a male‐specific allele that was consistently present in all males and absent in females. We then developed a Droplet Digital PCR (ddPCR) assay to quantify allele ratios and validated it on mock (DNA extract mixtures) and eDNA samples with known sex ratios. Our sex‐specific SNP assay successfully distinguished male‐ and female‐biassed ratios in mock samples and showed a strong positive relationship between the proportion of males and the male‐specific allele. While resolution was lower in eDNA samples, sex ratio estimates reflected population composition, particularly when corrected for biomass. Performance was mainly influenced by inter‐individual variation in male allele copy numbers, but this effect diminished as the number of males increased, reflecting natural populations better. For effective field application, maximising nuclear eDNA recovery, validating marker specificity and accounting for species‐specific life history traits when sampling will be crucial. With further field validation, our eDNA‐based method could support large‐scale, non‐invasive sex ratio monitoring, offering valuable insights into species phenology and population dynamics to guide conservation efforts.

## 1 Introduction

Studying the demographic processes of natural populations is fundamental to understanding their dynamics, evolutionary trajectories and ecological interactions (Griffith et al. 2016). Population demographic information, particularly in relation to reproduction (e.g., birth/death rates, mating patterns and sex ratios) provides valuable insights into the health and viability of species populations (Godwin et al. 2020). One of the main factors influencing the reproductive success of individuals in a population is the adult sex ratio (Lee et al. 2011; Székely et al. 2014). Imbalanced adult sex ratios can increase competition in finding mates, trigger sexual aggression or parental desertion and result in lower rates of survival and reproduction, ultimately leading to population collapse (Le Galliard et al. 2005; Liker et al. 2015; Eberhart‐Phillips et al. 2018). Despite their importance, deviating sex ratios are often overlooked, which can significantly underestimate extinction risk rates, especially in small and vulnerable populations (Bessa‐Gomes et al. 2004; Melbourne and Hastings 2008; Lee et al. 2011). In light of the ongoing biodiversity loss driven by anthropogenic pressures (IPBES 2019) and the impact of these pressures on sex ratios (Carter and Hopkins 2022), there is a pressing need for efficient, scalable and non‐invasive methods to monitor sex ratios as early indicators of population decline.

Research on sex ratios may be frequently neglected due to the labour‐intensive, invasive and biassed nature of traditional monitoring methods. For example, sex ratio information is typically obtained visually through observation or capture, and this can introduce bias due to behavioural and phenotypic differences between sexes or individuals (Ancona et al. 2017). Moreover, capturing is invasive and can lead to injury or mortality (Woodruff et al. 2020; Brundu et al. 2023). Alternatively, for sexually monomorphic species, if sex is genetically determined, DNA‐based approaches can be used by analysing an individual's tissue, blood or swab sample (Handel et al. 2006; Ghorpade et al. 2012). However, this form of monitoring remains challenging especially for species that are protected or of cultural significance and require permits that take time and effort (Adams et al. 2019). Various studies have used non‐invasive genetic sampling approaches to sex individuals and estimate sex ratios by collecting traces such as faeces, hair or feathers (Banks et al. 2003; Costantini et al. 2008; Bonesi et al. 2013; Baumgardt et al. 2013). Yet, population‐level non‐invasive sampling remains elusive.

Environmental DNA (eDNA), a novel non‐invasive method of monitoring species and communities by analysing the genetic material present in an environmental sample such as water, soil or air, offers a promising alternative to traditional monitoring approaches (Didaskalou et al. 2022). eDNA collection has proven itself to be a cost‐ and time‐efficient method (e.g., Qu and Stewart 2019), allowing researchers to take more samples across space and time, and detect elusive, cryptic or rare species (Ruppert et al. 2019; Banerjee et al. 2022). Furthermore, eDNA facilitates monitoring in hard‐to‐reach habitats (e.g., subterranean waters; Gorički et al. 2017), is less affected by weather conditions when sampling (Thomsen and Willerslev 2015), does not require permits to sample individuals and transporting samples internationally is less regulated (Sigsgaard et al. 2020). However, most eDNA studies have focused on taxonomic identification using mitochondrial DNA (mtDNA), while obtaining population‐level information such as sex ratios requires nuclear DNA (nuDNA) which poses greater technical challenges.

A major challenge of obtaining sex ratios with eDNA is that nuDNA degrades faster and is present in lower copy numbers than mtDNA (Foran 2006; Sigsgaard et al. 2020). Additionally, differences in behaviour, metabolism and diet can influence eDNA shedding rates and misrepresent abundance or biomass metrics (Maruyama et al. 2014; Klymus et al. 2015; Stewart 2019). Yet, since nuDNA is present in equal copy numbers across cell types, it may prove more suitable than mtDNA for abundance estimates (Sigsgaard et al. 2020; Jo et al. 2022). Despite the challenges associated with nuDNA, recent eDNA studies have demonstrated its potential for retrieving population‐level genetic information (Jensen et al. 2021; Andres et al. 2021, 2023; Adams et al. 2023; van Kuijk et al. 2025). The use of shorter markers like single nucleotide polymorphisms (SNPs) (Børsting et al. 2013) paired with highly sensitive analytical methods such as Droplet Digital PCR (ddPCR) (Mauvisseau et al. 2019) could reduce genotyping errors that pose a concern in low DNA quality and quantity samples like eDNA (Taberlet et al. 1999; McKelvey and Schwartz 2004). Interestingly, various ecological studies have successfully used SNP markers to identify the sex of individuals from both tissue (Maroso et al. 2016) and environmental samples (browsed twigs; Nichols and Spong 2017), highlighting the potential of sex‐linked SNPs for assessing the sex ratios of populations through eDNA.

To date, it remains unclear whether eDNA can be used to reliably estimate a population's sex ratio. Therefore, in this study, we aim to present an eDNA‐based method for monitoring sex ratios by quantifying sex‐specific SNP alleles. First, we identify candidate sex‐linked SNPs through population genomic analysis of sexed individuals and follow by validating their specificity in DNA extracts derived from tissue. For quantification, we use Droplet Digital PCR (ddPCR, Bio‐Rad Laboratories) with a rare mutation detection assay. We then assess the method's suitability, applicability and sensitivity using mock (DNA extracts) and eDNA samples with known sex ratio while also determining the assay's limits of detection and quantification. For this study, we use crested newts as our model species due to their aquatic reproductive phase, making them well‐suited for eDNA sampling, and their conservation concern linked to widespread population declines. This proof‐of‐concept study provides insights into the potential of using eDNA to estimate population sex ratios in wildlife monitoring.

## 2 Materials and Methods

### 2.1 Study System

Like most other amphibians, crested newts (Family Salamandridae, genus *Triturus*) spend the breeding season in water where they lay eggs. Eggs develop into larvae and, after several months, these larvae undergo metamorphosis, subsequently remaining terrestrial until they become sexually mature and return to water. Their dependence on breeding ponds and general lack of long‐distance migration makes crested newts particularly vulnerable to local extinction and they are indeed considered some of Europe's fastest declining amphibian taxa (Edgar and Bird 2006). Habitat fragmentation, changes in water use, pollution, the introduction of non‐native species, as well as disease represent major conservation challenges (Wielstra 2019), demanding rapid monitoring approaches. The Balkan crested newt ( *Triturus ivanbureschi* ), our model species, is found in the south‐eastern Balkan Peninsula, representing one of seven crested newts (Wielstra and Arntzen 2016).

According to cytological studies, all *Triturus* newts have an XY determination system (Sims et al. 1984), but due to the large genome size, molecular genomic analyses have been challenging. To identify sex‐linked SNPs, we use restriction site associated sequencing (RADseq) data, a cost‐effective method for population genomic analyses which does not require a reference genome, making it ideal for identifying sex‐linked markers in non‐model organisms such as crested newts (Hohenlohe et al. 2012). Considering the *Triturus* XY determination system, our eDNA‐based method for sex ratio assessment was developed by identifying and measuring the concentration of a male‐specific allele relative to the allele common in both male and female newts.

### 2.2 Sex‐Specific SNP Assay Development and Validation

To identify sex‐linked SNPs, RADseq data from 28 female and 29 male morphologically sexed *Triturus ivanbureschi* (France, Babik, Cvijanović, et al. 2025) were analysed and processed with Stacks v2.66 (Catchen et al. 2013). Individuals in this dataset originated from Zli Dol (Pčinja district, Serbia, Figure S4). First, reads were demultiplexed and trimmed using the *process_radtags* module (default settings). To assign reads to loci, the *denovo_map.pl* pipeline was used with the following parameters: minimum stack depth (*M* = 3), maximum nucleotide mismatches allowed within stacks (*m* = 3), mismatches between sample tags when building the catalogue (*n* = 3), minimum percentage of individuals in a population required to process a locus for that population (*r* = 0.8) and a minimum minor allele frequency required to process a nucleotide site at a locus (*min‐maf* = 0.2). Considering that samples come from the same population and genomic divergence is not expected to be high, these parameters (low *M*, and *n* = *M*) were chosen as optimal (Paris et al. 2017). From this analysis, 19 SNP loci with male‐specific alleles were identified (Observed Homozygosity in females was 100%, Heterozygosity observed only in males), but only in two loci (L68 and L28981) was the male allele present in the majority of males (Observed Heterozygosity in males > 80%), and these were selected for further validation.

Primers and competitive probes with locked nucleic acids (LNAs) were designed for the two selected loci (L68, 88 bp; L28981, 75 bp) (Figure S1 and Table S1) using the Primer3 plugin in Geneious Prime (v2024.0), following ddPCR assay guidelines for rare mutation detection (Bulletin_6407, Bio‐Rad Laboratories, n.d.‐a, Chapter 2 and 5). The incorporation of LNA bases allows for shorter probes with higher melting temperatures (T_m_), enhancing both stability and specificity, and improving sensitivity to single‐base mismatches (Ugozzoli et al. 2004). To optimise assay conditions, a gradient ddPCR (conditions as in section 2.6 and annealing temperature range: 63°C–53°C) was first performed for a male and a female sample to determine the optimal annealing temperature, by ensuring good positive–negative droplet separation, as well as probe specificity (no amplification in females). Further validation was performed to confirm that the common allele was detected in both sexes, while the male‐specific allele was exclusively detected in males. This validation was conducted using tissue‐derived DNA from 15 females and 23 males of *T. ivanbureschi* , collected from two populations positioned away from the hybrid zone with other *Triturus* species (Wielstra et al. 2017). Individuals used in subsequent eDNA experiments (see 2.5) were also obtained from these two populations (Zli Dol and Brebevnica, Serbia, Figure S4). Because the genomic location and copy number status of our target loci are unknown (e.g., whether it is coding or non‐coding, single or multi‐copy), common and male allele marker concentrations were compared to other presumed single copy nuclear DNA markers (DN59906 and DN55850; Meilink et al. 2025, Table S1) targeting coding regions (Wielstra et al. 2019).

### 2.3 Mock Sex Ratio Samples

**FIGURE 1:**
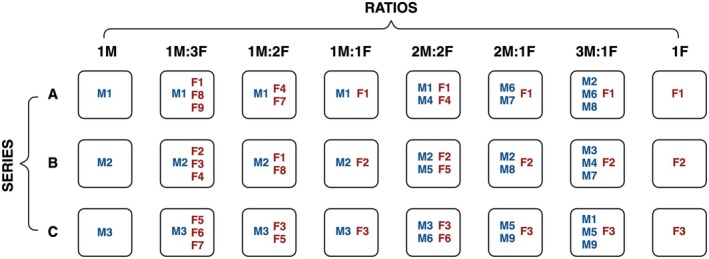
Representation of individuals ( *T. ivanbureschi* ) used to create the mock sex ratio samples. M denotes male and F denotes female. Each sample (square) was measured in triplicate. Created with BioRender.com.

The suitability of the selected sex‐linked SNPs for sex ratio estimation was evaluated using mock sex ratio samples created from 9 male and 9 female *T. ivanbureschi* DNA extracts that were randomly selected. DNA concentrations were first quantified in triplicate using the NanoQuant plate on a Spark Multimode microplate reader (Tecan), and the average value for each sample was used to normalise concentrations across individuals. Subsequently, mock sex ratio samples were created by systematically pairing different individuals. To assess the impact of individual variation in allele copy number, one male (used in the 1M:1F, 2M:2F, 1M:2F and 1M:3F ratios) and one female (used in the 1M:1F, 2M:2F, 2M:1F and 3M:1F ratios) were kept constant across replicate series (Figure 1). For each mock sample, 2 μL of DNA extract per individual was combined and the mixture diluted 1:10 prior to analysis. Each sex‐ratio treatment was replicated in three series with different individuals to assess individual variation (Figure 1), and each sample per series was analysed in triplicate (conditions as in section 2.6), including no‐template controls (NTCs).

### 2.4 Limit of Detection (LoD) and Limit of Quantification (LoQ)

To assess the sensitivity of the analytical method, we evaluated the lowest allele concentrations at which we could reliably detect the presence of the male allele and accurately estimate allele ratios. The Limit of Detection (LoD) was defined as the lowest concentration of the male allele that shows a statistically significant difference from a negative (female) sample containing only the common allele (Bulletin_6628, Bio‐Rad Laboratories, n.d.‐b, Chapter 1). Similarly, the Limit of Quantification (LoQ) was defined as the lowest concentration of the male allele at which allele ratios can be measured with acceptable precision. Since the LoQ depends on the level of uncertainty considered acceptable for a method's intended use (Deprez et al. 2016) and considering that eDNA samples typically contain low nuclear DNA concentrations that are more prone to stochastic variation, we set the maximum acceptable measurement uncertainty at 30%. To determine both LoD and LoQ, we measured concentrations of the common and male alleles using a 4‐fold dilution series of a male and a female DNA sample (conditions as in section 2.6). Dilutions included 1:4, 1:16, 1:64 and 1:256, starting from 1:10 diluted DNA extracts measured at 21.4 ng/μL (male) and 24.8 ng/μL (female). Each dilution, along with the starting sample and an NTC, were analysed in six replicates.

At very low concentrations, random partitioning of molecules among droplets can cause substantial variability between wells. To minimise this stochastic effect and enable reliable quantification at lower concentrations, the LoD and LoQ were calculated from the six replicates merged into one well. Merging allows the Poisson correction to be applied to the total droplet population, improving the precision of concentration and ratio estimates and permitting quantification at concentrations lower than those achievable from single wells (Bulletin_6407, Bio‐Rad Laboratories, n.d.‐a). Therefore, the LoD and LoQ values reported here reflect the precision attainable from merged replicate data and do not represent the detection or quantification limits of single‐well measurements. Given that eDNA samples often contain low nuclear DNA concentrations, it is advisable to include multiple technical replicates for such samples to enable lower limits of detection and quantification.

### 2.5 eDNA Experiments and Extractions

To determine the applicability of using eDNA sampling for the developed sex ratio estimation method, we conducted controlled experiments. Here, *T. ivanbureschi* individuals were placed in plastic containers filled with water under various sex‐ratio combinations, and eDNA samples were subsequently collected (Figure S3). These experiments were performed in a previously established breeding colony at the Institute for Biological Research ‘Siniša Stanković’ (University of Belgrade, Serbia). At the end of March 2023, all newts were introduced into large outdoor containers (500 L) that simulate natural aquatic conditions. Genetically pure *T. ivanbureschi* originated from two populations: Zli Dol (permit no. 353‐01‐75/2014‐08 of the Ministry of Energy, Development and Environmental Protection of the Republic of Serbia) and Brebevnica (permit no. 353‐01‐1506/2022‐04 of the Ministry of Environmental Protection of the Republic of Serbia) (Figure S4). The experimental procedures were approved by the Ethics Committee of the Institute for Biological Research ‘Siniša Stanković,’ University of Belgrade (decision no. 323‐07‐07479/2023‐05). All experimental animals were treated in compliance with the European directive (2010/63/EU) on the protection of animals used for experimental and other scientific purposes.

Three days prior to the experiment (June 2023), females and males were separated in different containers (200 L) to prevent contamination. To associate mass to eDNA concentrations, all individuals were weighed (g) prior to the experiments (Table S6). The sex ratio combinations applied were the same as in the mock sample experiment, including blank containers with no newts; all treatments (sex ratios) were replicated 3 times with different individuals (Figure S2). Due to the limited number of *T. ivanbureschi* individuals, every replicate of each treatment (Figure S2) was performed on a different day, and individuals were used more than once, ensuring they were randomly assigned to containers. For the experiment, 45 L plastic containers were used. Before use and between each swap, all containers (including blank) were decontaminated with a 0.05% sodium hypochlorite household bleach solution to ensure sterilisation without negatively affecting the health of the newts. Containers were then thoroughly rinsed with tap water to remove any residual bleach and subsequently filled to the top with tap water. Approximately 24 h prior to introducing the newts, all containers (including blanks) were emptied and refilled with 24 L of fresh tap water, which was left to dechlorinate for 24 h. To avoid potential contamination with male DNA, females were always handled before males. After placement in the containers, three *Musca domestica* larvae were provided per individual for feeding. A 72‐h acclimation period was implemented to minimise fluctuations in eDNA concentration in case of stress due to handling. Following this period, eDNA samples were collected by filtering 800 mL of water from each container using a self‐preserving eDNA filter pack (Smith‐Root) fitted with a 0.45 μm pore‐size PES membrane. Filtration was performed using an eDNA citizen scientist sampler (Smith‐Root), and filters were fully dried before storage by allowing the pump to run in air. All filters were stored and transported at room temperature until further processing.

Filters were processed at Leiden University 3 days after the end of experiments in Serbia, in a dedicated pre‐PCR laboratory under a DNA/RNA UV‐cleaner box (UVT‐B‐AR, Biosan). The filters were cut in half with scissors, and each half was stored in separate 2 mL tubes with 360ul ATL buffer (Qiagen) and further stored in –20°C for 9 months until DNA extraction. Tweezers and scissors used to process filters were decontaminated in a 0.5% sodium hypochlorite household bleach solution and rinsed in a series of 3 clean demi water solutions that were UV treated for 20 min. Half filters were extracted with a modified Qiagen DNeasy Blood & Tissue kit protocol (Spens et al. 2017) including a QIAshredder (Qiagen) column after lysis and two extraction blanks. After extraction, samples were stored at –20°C until further analysis.

### 2.6 Droplet Digital PCR (ddPCR)

To estimate sex ratios, the concentration of the common and male alleles was measured using the QX200 Droplet Digital PCR (ddPCR) system from Bio‐Rad Laboratories. The duplex ddPCR reaction mixture (22 μL) consisted of 11 μL of ddPCR Supermix for Probes (1863024, Bio‐Rad Laboratories), 227 nM of each TaqMan probe, 455 nM of each primer, 4 μL of DNA template and UltraPure DNase/RNase‐Free Distilled Water (Invitrogen) up to 22 μL. For the gradient, validation and mock ratios we used 2 μL of DNA template. The PCR reaction was partitioned into droplets using the QX200 Droplet Generator (Bio‐Rad Laboratories) and then amplified using a thermal cycler (T100 Touch, Bio‐Rad Laboratories). Initial denaturation was performed at 95°C for 10 min, followed by 40 cycles at 94°C for 30 s and at 57°C for 1 min, followed by enzyme deactivation at 98°C for 10mins and a hold at 4°C, all with a ramp rate of 2°C/s. After amplification, the droplets were analysed using the QX200 Droplet Reader (Bio‐Rad Laboratories). Threshold values for determining positive droplets were determined manually per plate (see section 3.1) using the QuantaSoft software (version 1.7; Bio‐Rad Laboratories). NTCs were used as negative controls to test for contamination during sample preparation. eDNA samples were measured in triplicate twice in different plates. To calculate merged concentrations from the six replicates, the concentration equation as described on the ddPCR applications guide was used (Bulletin_6407, Bio‐Rad Laboratories, n.d.‐a, Chapter 3). Before analysis, outputted copies/μL were corrected to copies/μL in the starting material by multiplying with the total volume of the ddPCR mix (22 μL) and then dividing by the volume of sample used (2 or 4 μL).

### 2.7 Data Analysis

To determine the LoD of the male allele, copy number concentrations were compared with those from negative controls containing only the common allele (female). When copy number concentrations from replicate samples of the male allele showed overlapping confidence intervals with those from negative controls (female sample), concentrations were compared using *t*‐tests (function: ‘t.test’, R package: *stats*). Because the negative controls consisted entirely of zero values, a one‐sample *t*‐test was performed to test whether the mean concentration of the male allele differed significantly from zero. Data normality of the male sample was verified using a Shapiro–Wilk test (function: ‘shapiro.test’, R package: *stats*). The LoD was set at the lowest concentration that yielded a significant difference from negative controls. Our LoQ was determined as the lowest copy number concentration of the male allele from which common‐to‐male ratios exhibited a relative standard deviation of < 30%.

To test whether the common‐to‐male allele ratios of the different male‐to‐female ratio groups for the mock samples differed significantly, we performed a one‐way ANOVA (function: ‘aov’, R package: *stats*) followed by a Tukey's Honestly Significant Difference (HSD) test to control the family‐wise error rate (function: ‘TukeyHSD’, R package: *stats*). Assumptions of normality of model residuals and heteroskedasticity were checked based on residual plots (function: ‘qqPlot’ and ‘leveneTest’, R package: *car*, Fox and Weisberg 2018; function: ‘hist’, R package: *graphics*). When assumptions for normality were violated, common‐to‐male allele ratios were Log_10_‐transformed, and models were rechecked. A linear regression was subsequently performed to evaluate the relationship between the ratio of the male‐to‐common allele and the ratio of male‐to‐total individuals in mock samples (function: ‘lm’, R package: *stats*). Assumptions of normality of model residuals were checked, and transformations (Log_10_ + 1) were performed when needed, as described for one‐way ANOVAs. Models were also compared using the Akaike information criterion (function: ‘AIC’, R package: *stats*).

To assess the quantitative accuracy of the assay, we compared our observed common‐to‐male allele ratios from mock samples with expected hypothesised (average) values in males (7:1, see section 3.1). For each sex ratio group, one‐sample Wilcoxon signed‐rank tests (function: ‘wilcox. test’, R package: *stats*) were performed to test whether observed ratios significantly differed from the expected values. In addition, a linear regression was performed (as described above, but with a Log_10_ transformation instead) to assess the overall relationship between observed and expected common‐to‐male allele ratios across all groups. A slope not significantly different from one was interpreted as evidence of accurate quantification (function: ‘confint’, R package: *stats*, function: ‘linearHypothesis’, R package: *car*).

To determine the applicability of the developed method to eDNA samples, the same approach was applied as described for the mock samples. Three datapoints (one for group 1M, one for group 1M:1F, and one for group 1M:3F) were excluded from our analysis as these did not fall within the LoD and LoQ derived above. These samples showed low concentrations of the common allele as well, suggesting limited total DNA input. To determine whether this reflected generally low newt DNA or issues specific to the nuclear marker, mtDNA concentrations were quantified with marker ND4 (Table S1). While mtDNA concentrations were indeed low in these samples, other samples with similarly low mtDNA still exhibited higher concentrations of both common and male alleles (Table S4). Since data transformation for the eDNA dataset did not yield normally distributed model residuals, a non‐parametric Kruskal‐Wallis test was performed (function: ‘kruskal.test’, R package: *stats*), followed by a Dunn's test (function: ‘dunnTest’, package: *FSA*, Ogle et al. 2025) using the Benjamini‐Hochberg method. To account for potential variation in eDNA shedding related to individual and sex‐based differences in body mass, eDNA data were mass‐corrected by multiplying the observed allele ratios with the proportion of male‐to‐total mass (for male‐to‐common allele ratios) or total‐to‐male mass (for common‐to‐male ratios, Table S7) and reanalysed as described above. All statistics were performed using R version 4.3.1 (R Core Team 2021), with additional packages *car* (Fox and Weisberg 2018) and *FSA* (Ogle et al. 2025). Test outcomes were considered statistically significant at *p* < 0.05.

## 3 Results

### 3.1 Sex‐Specific SNP Assay Validation

The gradient ddPCR results revealed a slight cross‐reactivity of the male allele probe, with non‐target DNA in females, yielding 1–2 droplets across all tested temperatures (53°C–63°C). However, the fluorescence amplitude of these signals remained below 2000 (HEX), whereas the target male allele consistently showed amplitudes between 3000 and 4000 (HEX). This difference was considered sufficient to prevent false positive detection. To account for this minor cross‐reactivity, a conservative fluorescence threshold of minimum 2400 HEX amplitude was applied in all subsequent measurements and for the common allele the threshold was set to a minimum of 3000 FAM amplitude (Figure S5).

Common alleles for both loci (L68 and L28981) were detected in all individuals, while, as expected, male alleles were male‐specific (i.e., absent from all tested female samples). The male allele for locus 68 (L68) was present in all tested males, but the male allele for locus 28981 (L28981) was absent in 3 out of 23 males. Ratios of common‐to‐male allele for both loci varied per individual and ranged between 3:1 and 14:1 (Table S2). One male sample was excluded from quantification for L68 due to disproportionately high DNA concentration, resulting in an excessive number of droplets between positives and negatives, making the threshold unreliable. The average common‐to‐male allele ratio for both L68 and L28981 was 7:1, but the relative standard deviation (RSD) for L28981 was larger; RSD for L68 was 38% and for L28981 48%. Given that the male allele for L28981 was not consistently detected in all males, all subsequent analyses focused on L68. Lastly, concentrations of the common and male alleles for L68 were higher across individuals than nuclear DNA markers targeting coding regions (Table S5).

### 3.2 Limit of Detection (LoD) and Limit of Quantification (LoQ)

Measurements of the serial dilutions yielded positive droplets for the male allele in 4 out of 6 replicates (6 positive droplets in total) at the lowest dilution (8.4 pg/μL or 1:256) of the male sample (Table S8), corresponding to a concentration of 0.39 copies/μL (merged replicates), whereas the female sample at the same dilution had zero positive droplets (Table 1). Although the 95% confidence intervals of the male and female samples overlapped (male: 0.11–0.77 copies/μL; female: 0–0.17 copies/μL), a one‐sample t‐test revealed that the mean male allele concentration was significantly different from zero (t = 2.6, df = 5, *p* = 0.047), and as such the male sample at the lowest dilution was considered a true positive (Bulletin_6628, Bio‐Rad Laboratories, n.d.‐b, Chapter 4). Accordingly, the LoD for the male allele was defined as 0.39 copies/μL at 8.4 pg/μL (i.e., the lowest concentration present in the serial dilution) provided that a minimum of 6 replicates was analysed. Although no positive droplets for the male allele were observed in any of the diluted female samples, the original undiluted female sample (2480 pg/μL) yielded positive droplets in 3 of 6 replicates (4 droplets total), even when applying a conservative threshold (Table S8). However, these were below the defined LoD and did not affect further analyses. All no‐template controls (NTCs) were negative for both the common and male allele.

**TABLE 1:** Concentration (copies/μL) of L68 common and male allele, their ratio (common‐to‐male) and the percentage of relative standard deviation (RSD%) from replicate ratio measurements per dilution. Concentrations are generated from 6 replicates merged into one well. Ratios were calculated by the ddPCR software using Poisson statistics across all merged wells.

Dilution	Male				Female	
	Common	Male	Ratio	RSD%	Common	Male
1	685.85	82.50	8.33	12.11	768.90	0.25
1:4	172.15	20.52	8.40	10.26	180.95	0.00
1:16	41.25	6.49	6.40	39.96	43.45	0.00
1:64	8.36	1.16	7.30	66.06	10.07	0.00
1:256	2.42	0.39	7.00	85.24	2.31	0.00

Regarding the LoQ, the uncertainty (i.e., relative standard deviation between replicates) in ratio calculations of replicates exceeded 30% from dilutions 1:16 (113.8 pg/μL) onwards (Table 1). This is in line with expectations, since the subsampling error is inherently more significant at lower concentrations. When replicates were merged, variation in ratio measurements across dilutions was considerably lower, with a relative standard deviation of 12% between dilution measurements. This suggests that with sufficient replication (≥ 6 replicates), reliable ratio estimates within the target variance threshold could still be achieved at the lowest tested concentration (8.4 pg/μL or 1:256). Based on this, the LoQ was defined at 0.39 copies/μL provided that a minimum of 6 replicates is measured.

### 3.3 Mock and eDNA Sex Ratio Samples

**FIGURE 2:**
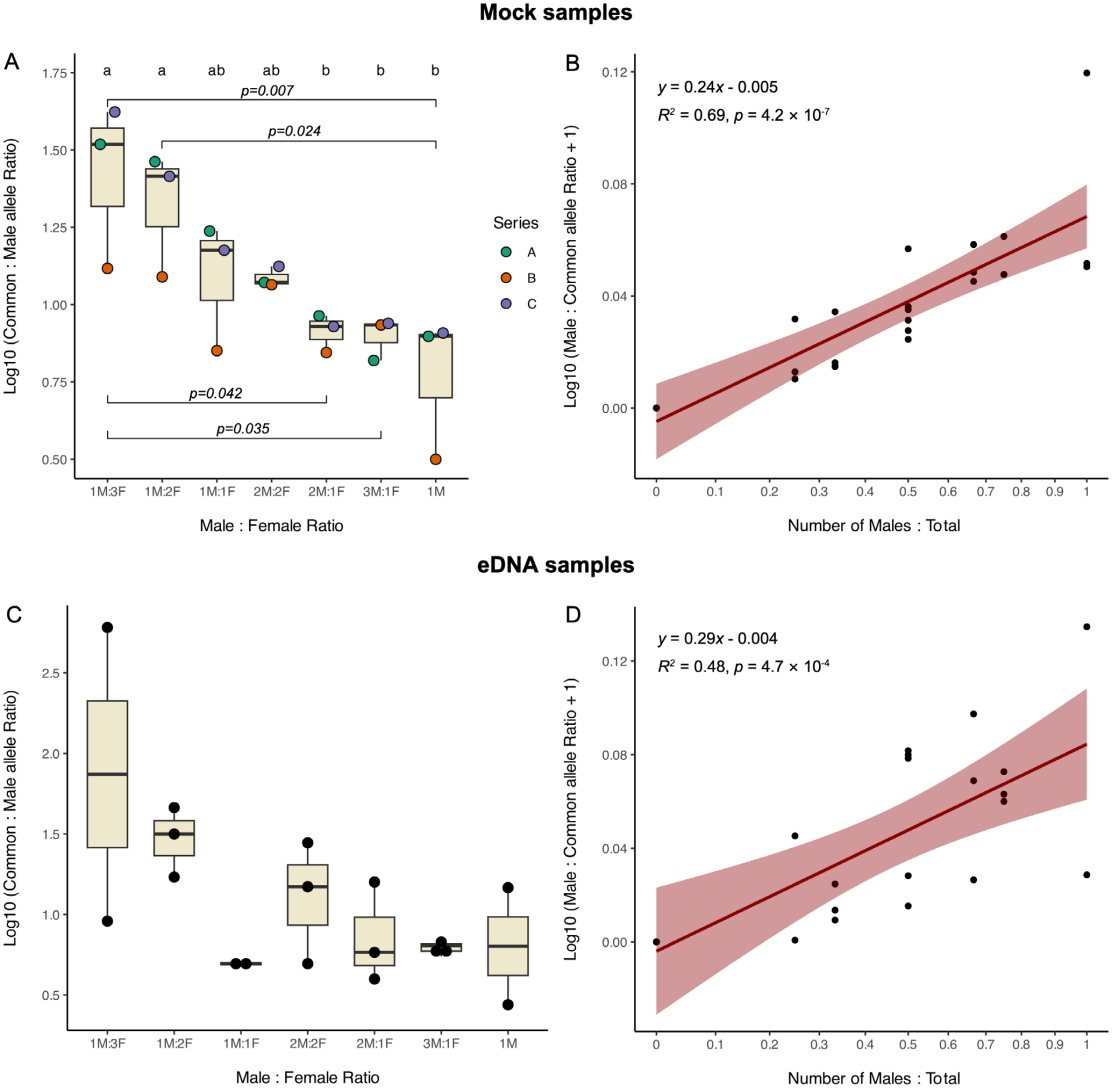
(A, C) Log_10_‐transformed ratios of common‐to‐male allele concentrations (copies/μL) across different male‐to‐female ratios in mock (A) and eDNA (C) samples. Boxplots show medians, interquartile ranges and individual data points. Letters above boxes indicate groupings based on Tukey's HSD post hoc tests which adjust *p*‐values for multiple testing using the Studentised range distribution; horizontal bars denote significant pairwise comparisons (*p* < 0.05). (B, D) Linear regressions of Log_10_ (male‐to‐common allele ratio + 1) against the proportion of males in mock (B) and eDNA (D) samples. Both variables were Log_10_ + 1 transformed for analysis; *x*‐axes are back‐transformed to raw scale for interpretability. Shaded ribbons represent 95% confidence intervals.

Allele ratio patterns for the mock samples reflected the expected sex composition. Common‐to‐male allele ratios decreased as the male proportion increased, with a significant overall difference among groups (*F*_(6)_ = 5.3, *p* = 0.005, Figure 2A). Post hoc tests revealed that samples with female bias differed significantly from male‐dominated groups, demonstrating the assay's ability to detect differences in sex ratio composition between groups. Additionally, variability within groups was mainly driven by Series B, pointing out the effect of individual differences in allele copy numbers. Common allele concentrations were consistent across mock samples (Table S3), indicating that the main cause of variation observed in allele ratios is likely due to differences in the copy numbers of the male allele. Notably, a specific male sample (M2, Figure 1) had a distinctly lower common‐to‐male allele ratio, which disproportionately influenced the results of the mock sex ratio samples with one male, but this effect diminished in mock samples containing multiple males (Figure 2A). The quantitative performance of the assay was supported by a strong linear relationship of male‐to‐common allele ratios against the proportion of males in the sample (*R* ^2^ = 0.69, *F*_(19)_ = 35.1, *p* = 4.2 × 10^−7^, Figure 2B), suggesting the assay's further ability to estimate male proportion in a continuous manner. All‐female mock samples tested negative for the male‐specific allele, and NTCs were negative for both common and male alleles, confirming assay specificity and absence of contamination. Furthermore, no significant deviations between observed and expected (hypothesised) common‐to‐male allele ratios were detected (*p* > 0.05 for all groups, Table S9), and the linear regression between observed and expected common‐to‐male allele ratios revealed a strong positive relationship (*R* ^2^ = 0.69, *F*_(19)_ = 41.9, *p* = 3.3 × 10^−6^) with a slope not significantly different from one (*β* = 1.13, 95% CI = 0.77–1.50, *p* = 0.46), indicating that the assay reflected the known sex ratios in the mock samples.

Patterns for the eDNA samples were more variable and did not reveal significant differences in common‐to‐male allele ratios across sex ratio groups (*χ* ^2^_(6)_ = 9.3, *p* = 0.156), suggesting limited resolution for detecting categorical sex ratio differences from eDNA samples. However, the regression analysis of male‐to‐common allele ratios (Figure 2D) showed a significant linear relationship with the proportion of males (*R* ^2^ = 0.48, *F*_(16)_ = 7.5, *p* = 4.7 × 10^−4^), indicating that quantitative detection remains feasible. As in the mock samples, no male‐specific alleles were detected in all‐female eDNA tanks, and all eDNA controls, extraction blanks and NTCs were negative for both alleles.

**FIGURE 3:**
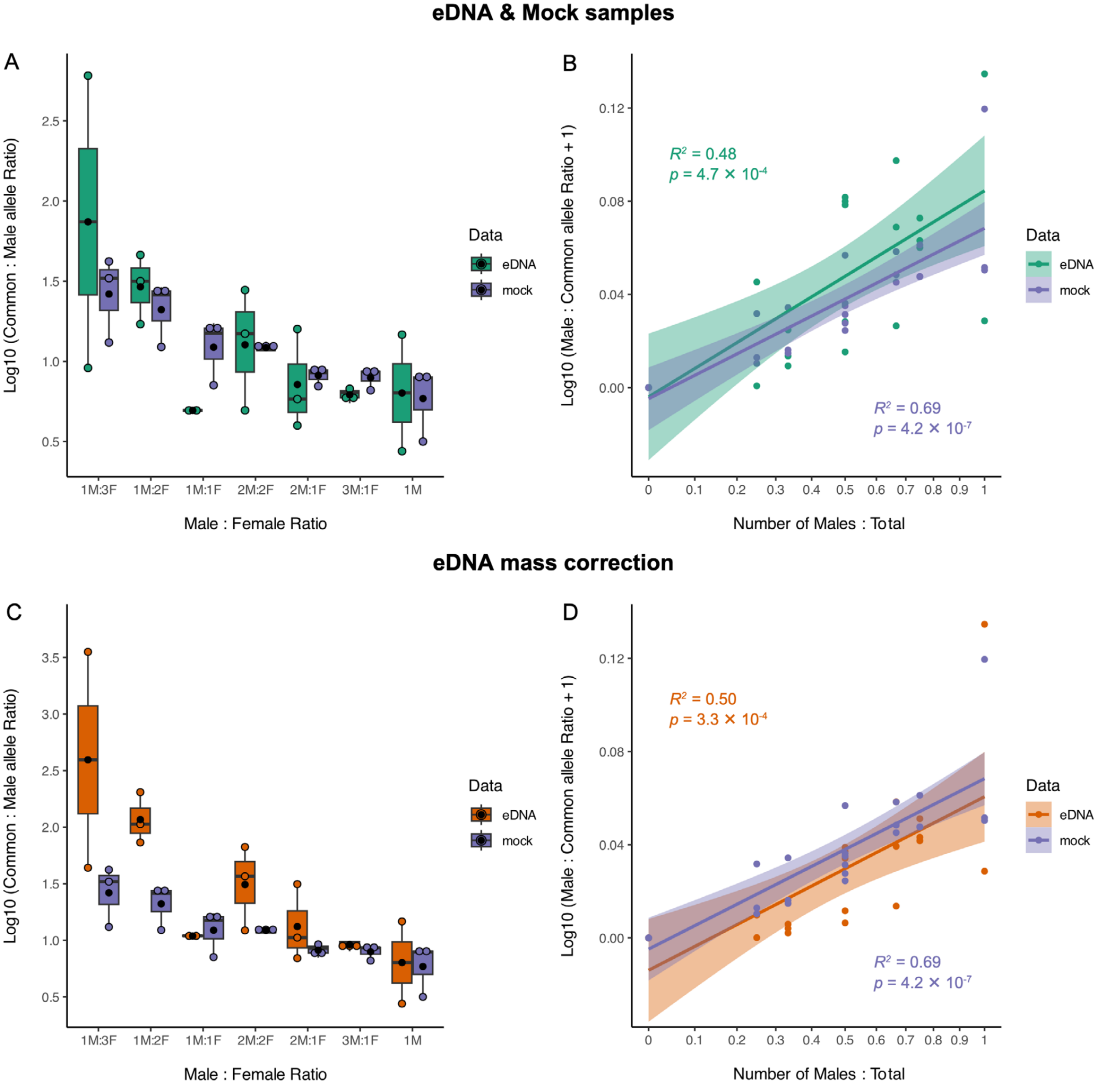
(A, C) Log_10_‐transformed ratios of common‐to‐male allele concentrations (copies/μL) across different male‐to‐female ratios in (A) mock and eDNA samples and (C) mock and mass‐corrected eDNA samples. Boxplots show medians, interquartile ranges and individual data points. Black dots indicate the mean. (B, D) Linear regressions of Log_10_(male‐to‐common allele ratio + 1) against the proportion of males in (B) mock and eDNA samples and (D) mock and mass‐corrected eDNA samples. Both variables were Log_10_ + 1 transformed for analysis; *x*‐axes are back‐transformed to raw scale for interpretability. Shaded ribbons represent 95% confidence intervals.

While variability was greater in eDNA samples compared to mock samples, the overall patterns remained consistent across both datasets (Figure 3A,B). Common‐to‐male allele ratios consistently decreased with increasing male proportion, and male‐to‐common allele ratios increased proportionally with the number of males, mirroring the trends observed in mock samples. After applying mass correction to the eDNA dataset, patterns became slightly more distinct (Figure 3C) and revealed a significant effect of sex ratio groups on common‐to‐male allele ratios (*χ* ^2^_(6)_ = 12.6, *p* = 0.049). However, post hoc pairwise comparisons did not detect any statistically significant differences between groups. The strength of the linear relationship between male‐to‐common allele ratio and male proportion also improved (*R* ^2^ = 0.50, *p* = 3.3 × 10^−4^, Figure 3D).

## 4 Discussion

Our study demonstrates the potential of environmental DNA (eDNA) for assessing sex ratios from populations. We achieved this by: (1) identifying and validating a male‐specific allele, consistently present in all tested males and absent in all females; (2) developing a sensitive ddPCR assay that can detect and quantify allele ratios at low DNA concentrations; (3) validating the performance of the assay for sex ratio estimation using mock sex ratio samples (DNA extract mixtures), which showed consistent and predictable shifts in allele ratios across different male‐to‐female ratios and (4) demonstrating that allele ratios from eDNA samples reflect sex ratios in controlled eDNA experiments with known male‐to‐female ratios. These results confirm the potential of using eDNA to estimate sex ratios in natural populations, providing a non‐invasive tool for ecological and conservation research.

### 4.1 eDNA‐Based Sex Ratio Estimates

eDNA‐based sex ratio estimates can reflect population composition, particularly when accounting for biomass. While previous studies used non‐invasive samples from traces of individuals to estimate sex ratios (Banks et al. 2003; Costantini et al. 2008; Bonesi et al. 2013; Baumgardt et al. 2013), none have tried to estimate a population average through non‐invasive samples such as water, soil or air. Our results demonstrate that, despite the greater variability, eDNA samples follow the same trends as mock samples and while resolution was too low to detect qualitative differences across different sex ratio groups, quantitative detection remained feasible. When allele ratios were corrected for biomass, patterns became even more distinct and statistical significance increased. This suggests that eDNA‐based sex ratio estimates may be more reliable when scaled by biomass rather than by individual counts, particularly in species where body size or behaviour differs between sexes. For instance, in crested newts, females are generally larger than males (Arntzen 2000). This correlation between eDNA concentration and organismal biomass has been demonstrated in previous studies (Pilliod et al. 2013; Doi et al. 2017; Salter et al. 2019), indicating that biomass plays a key role in an individual's eDNA contribution. However, the relationship between biomass and eDNA shedding may not be strictly linear; larger individuals may contribute disproportionately more or less eDNA than smaller ones (Yates, Glaser, et al. 2021). Therefore, applying allometric scaling, which accounts for these non‐linear relationships between body size and biological rates, may offer a more accurate approach for interpreting eDNA‐based estimates in future studies (Yates, Glaser, et al. 2021; Yates, Wilcox, et al. 2021; Urban et al. 2023).

### 4.2 Influence of Copy Number Variation on eDNA‐Based Sex Ratio Estimates

Our results suggest inter‐individual variation in male allele copy numbers can challenge sex ratio accuracy derived from eDNA samples, but dynamics in natural populations likely buffer this effect. While in mock samples SNP marker L68 was able to distinguish between male and female biassed ratios both qualitatively and quantitatively, the effect of inter‐individual variation in male allele copy numbers was strong in groups with a single male. In contrast, when the number of males increased, this variation diminished. This suggests that individual variation is averaged out when multiple males contribute to the eDNA pool (usually the case in natural populations) and improves the reliability of sex ratio estimates. Supporting this, ratio group 3M:1F in eDNA samples showed the least variability, further reinforcing the idea that this inter‐individual variation will be averaged out even in moderately sized populations. Unfortunately, due to the limited number of crested newts available, it was not possible for us to perform experiments with larger numbers per container.

Helping to mitigate inter‐individual variation in allele copy number could be an approach that targets coding regions for marker development, but this presents its own challenges for eDNA applications. While copy numbers in coding regions are typically more conserved (Redon et al. 2006), this approach is often limited due to the lack of annotated sex‐specific genes in many non‐model species, including *T. ivanbureschi* . In our study, the common and male alleles for L68 were more abundant across individuals than presumed single copy nuclear DNA (nuDNA) markers targeting coding regions, which improved detection rates in our eDNA samples but may also suggest L68 was not single copy (at least across individuals). Thus, although markers in coding regions may be less likely to exhibit variable copy numbers, their low abundance might hinder eDNA detection altogether. Salamanders, for instance, have a very large genome with extensive repetitive DNA (Sun et al. 2012), so certain genomic regions may occur at unexpectedly high copy numbers. In a previous study on the common carp, Minamoto et al. (2017) demonstrated that non‐coding multi‐copy nuDNA markers, such as internal transcribed spacer (ITS) regions, can occur at higher copy numbers than mtDNA markers and can therefore increase eDNA detectability. However, copy numbers in ribosomal DNA genes (including their ITS regions) can vary within species or populations (Hall et al. 2022). Similarly, studies on mammalian Y chromosomes have shown that male‐specific regions are maintained by gene conversion between mirrored sequences (palindromes), which can lead to copy number variation across individuals (Skaletsky et al. 2003; Rozen et al. 2003). Therefore, depending on their genomic location, copy numbers of sex‐specific markers (coding and non‐coding regions) may vary. This highlights that there may be a trade‐off between marker consistency and eDNA detectability which must be considered when selecting target regions; incorporating multiple sex‐specific markers may help mitigate these challenges.

### 4.3 Defining Analytical Thresholds for eDNA‐Based Sex Ratio Estimation

Establishing clear analytical methods is key for reliable eDNA‐based sex ratio estimation, particularly given the low concentrations and heterogeneous distribution of nuDNA in environmental samples. Despite the higher detection rates of marker L68 and our thorough technical replication (6 replicates), we excluded three samples from our eDNA analysis because the male allele concentration fell below the established LoD. Although both nuDNA and mtDNA concentrations were low in these samples, other samples with similarly low mtDNA exhibited higher concentrations of both common and male alleles, suggesting that eDNA heterogeneity (Troth et al. 2021; Urabe et al. 2025) and/or faster degradation of nuDNA (Foran 2006; Jo et al. 2022) could be contributing factors to this variation. Furthermore, our LoD was defined based on the lowest tested dilution, but it could be in fact lower. By merging multiple wells (replicates) into one meta‐well, ddPCR provides more flexibility to adjust the LoD by adjusting the number of wells screened (Bulletin_6407, Bio‐Rad Laboratories, n.d.‐a). Consequently, by increasing replication of samples, as well as negatives (female) to ensure assay specificity and NTCs to ensure no contamination, we could decrease the LoD further.

In contrast, for the LoQ, a more conservative approach is needed. While the serial dilution demonstrated that reliable ratio estimates are achievable at low concentrations with sufficient replication, the inherent low abundance of nuDNA in the environment introduces additional subsampling error. Establishing a concentration threshold for the common nuclear marker would help define cases where ratio estimates become unreliable, or where non‐detection of the male marker cannot be confidently interpreted as a true absence. A practical approach could be to first measure mtDNA concentrations to assess overall eDNA abundance and then proceed with sex ratio estimation only for samples likely to contain sufficient nuDNA. Samples with low male marker concentrations or no male detection can then be prioritised for additional replication to improve confidence in ratio estimates or detection. Ultimately, to obtain robust sex ratio estimates from eDNA, optimising replication effort or water volume filtered will be essential.

### 4.4 Considerations for Field Application

We envision that effective field application of our eDNA‐based method would require careful marker validation and sampling strategies that account for species‐specific life history traits. For reliable sex ratio estimates in the field, aside from enhancing the recovery of nuclear eDNA, it would also be crucial to evaluate the species specificity of the marker, particularly in populations where closely related species are sympatric. However, due to genetic variation, sex‐specific SNPs may not be conserved across closely related species or populations (Salisbury et al. 2003; Shi et al. 2018), which stresses the importance of validating sex‐specific markers in each target population before field application. Alternatively, including individuals from multiple populations during marker discovery can improve marker transferability. In populations with multiple life stages, translating biomass‐based estimates back into individual counts becomes more challenging, even when the average mass of each sex is known, and depending on the phenology of the species, the timing of eDNA sampling is critical to accurately capture relevant sex ratio dynamics (e.g., adult, larval or mixed cohorts). Future work could address this by incorporating age‐class information through eDNA methylation pattern analysis (Zhao et al. 2023) or by measuring the expression of life‐stage‐specific genes using environmental RNA (eRNA) approaches (Parsley and Goldberg 2024). In addition, behavioural differences between sexes like sex‐specific habitat use or phenology, as well as species‐specific reproductive strategies such as external fertilisation, may bias sex ratio estimates and should be considered during sampling design or when interpreting eDNA‐based sex ratio data. While our eDNA approach estimates sex based on genotype, in taxa where sex reversal is common, integrating eRNA would be essential to differentiate genotypic and phenotypic sex. Yet, our limited knowledge of sex‐specific genes in non‐model organisms and the type of tissues they are expressed in (Stevens and Parsley 2023) is currently restricting eRNA approaches from progressing in this direction. Instead, identifying sex‐linked SNPs or sequences with methods like RADseq (Trenkel et al. 2020; France, Babik, Dudek, et al. 2025) is a more accessible alternative for non‐invasive sex ratio estimation.

## 5 Conclusion

Our study is the first to demonstrate the potential of eDNA for assessing sex ratios in natural populations. Our sex‐specific SNP assay distinguished male‐ and female‐biassed ratios in mock samples and showed similar qualitative patterns in eDNA samples, especially after correcting for biomass. Both sample types exhibited a strong positive relationship between the proportion of males and the male‐specific allele, suggesting the method could support quantitative estimates from eDNA samples with increased nuDNA recovery. Variation was largely driven by differences in individual male allele copy numbers, though this effect diminished with multiple males. With further field validation and by establishing the sample volumes or replication required, this approach could enable accurate, large‐scale and non‐invasive sex ratio monitoring. Such scalable methods offer valuable insights into species ecology, phenology and population dynamics thereby supporting more effective conservation efforts.

## Author Contributions

E.A.D. and K.A.S. designed the study. E.A.D., K.B.T., P.M.B., K.A.S. conceptualised the study. J.F. and B.W. provided genomic data and DNA samples for validation. E.A.D., M.C., T.V., M.A. and A.I. prepared and performed the eDNA experiment. E.A.D. analysed all data. E.A.D. wrote the first draft of the manuscript. All authors contributed to editing the manuscript and approved the submitted version.

## Disclosure

Benefit‐sharing statement: Benefits from this research were generated via collaboration with scientists in Serbia who contributed to the eDNA experiment and shared their facilities. All data are publicly shared as described below.

## Conflicts of Interest

The authors declare no conflicts of interest.

## Supporting information


**Data S1:** men70089‐sup‐0001‐Supinfo.zip.

## Data Availability

The data that supports the findings of this study are available in the Supporting Information of this article.
