# Supplementary material for: Unlocking Demography: Developing an eDNA‐Based Toolkit to Measure Sex Ratios From Populations
Source: Mol Ecol Resour. 2025 Dec 18;26(1):e70089. doi: 10.1111/1755-0998.70089 (PMC12715386; doi:10.1111/1755-0998.70089)
Supplement: Supplementary file 1 — Data S1: men70089‐sup‐0001‐Supinfo.zip. [file MEN-26-e70089-s001.zip › men70089-sup-0002-FigureS1-S3-TableS1-S4@Suppl_info_Didaskalou_et_al._2025.docx]

**Supplementary Information**

**Supplementary Figures**


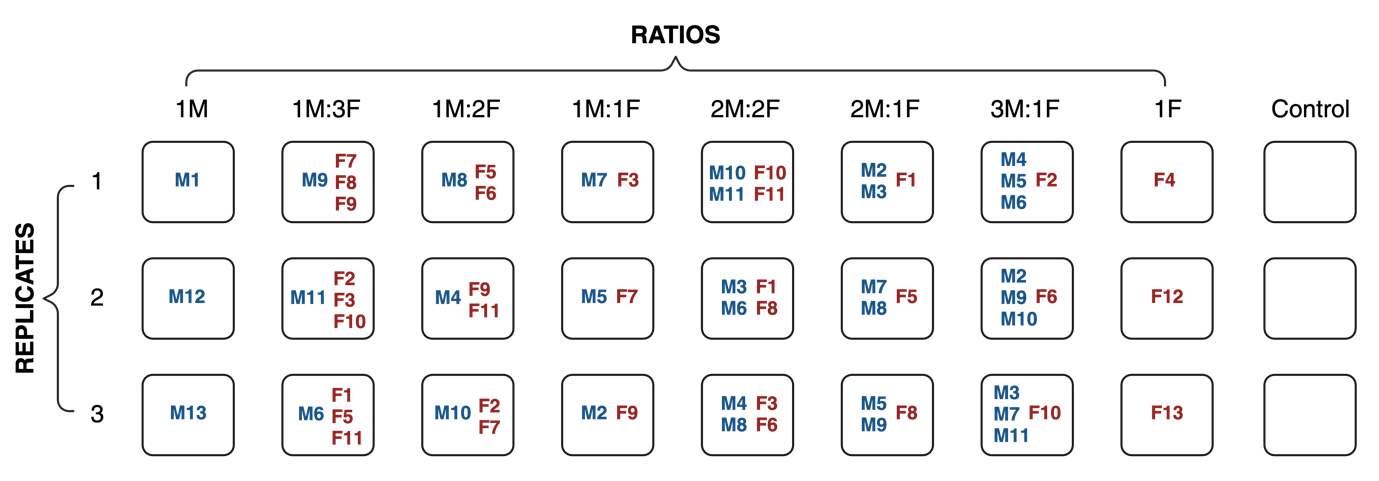


**Figure S1.** Representation of individuals (Triturus ivanbureschi) used in the eDNA sex ratio experiments. M denotes male and F female. Control treatment did not include any individuals.


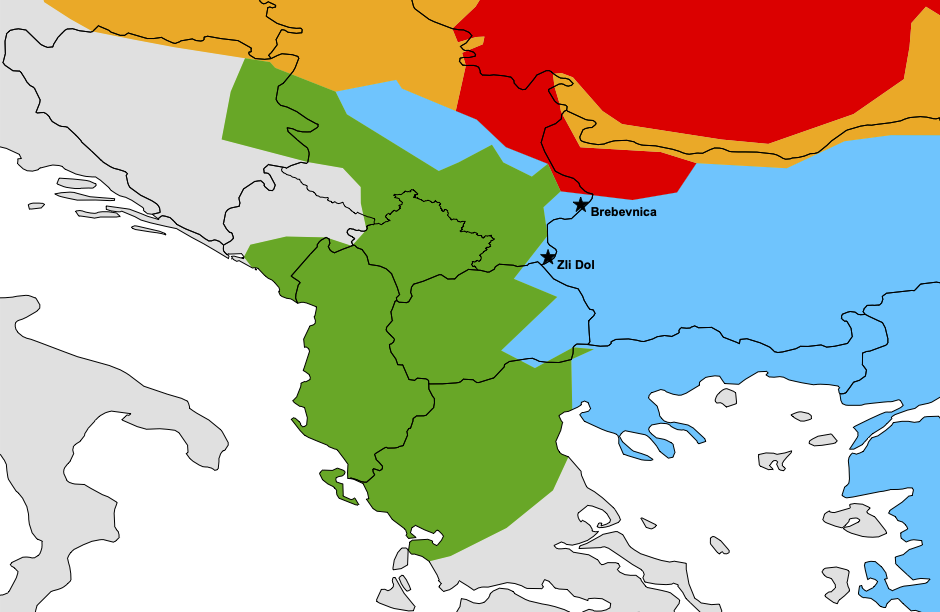


**Figure S3.** Map showing the distribution of *Triturus* species in the Balkans: *T. ivanbureschi* (blue), *T. macedonicus* (green), *T. cristatus* (red) and *T. dobrogicus* (orange). The stars represent the two locations in Serbia (close to Bulgarian borders) where individuals for this experiment originated in nature.

**Supplementary Tables**

**Table S1.** Sequences and modifications of primers and competitive probes for loci L68, L28981, mtDNA marker ND4 and nuclear markers targeting coding regions (DN59906 and DN55850; Meilink et al., 2025). The “+” signifies the location of locked nucleic acids (LNAs).

| **Oligo name** | **Sequence (5’ – 3’)** |
| --- | --- |
| L68 forward primer | GCTGCACATTACTCGACATATG |
| L68 reverse primer | TGAGAACAGAAGGCCTCG |
| L68 common allele probe | 6FAM – TGA+CATG+A+T+AGAAGA+CAA – IABkFQ |
| L68 male allele probe | HEX – TGACATG+A+C+AGAAGA+CAA – IABkFQ |
| L28981 forward primer | ATACAGTGATGCCTTTCG |
| L28981 reverse primer | ATTCATGCAACACCTCATTG |
| L28981 common allele probe | 6FAM – TGCGGCATT+T+C+AATTTTT – IABKFQ |
| L28981 male allele probe | HEX – TGCGG+CATT+T+A+AATT+TTT – IABkFQ |
| ND4 forward primer | CCTAGTCATCGCCGCTATT |
| ND4 reverse primer | TGATGAGATAAGCCCGTGTG |
| ND4 probe  DN59906 forward  DN59906 reverse  DN59906 probe  DN55850 forward  DN55850 reverse  DN55850 probe | 6FAM – ACACCGTTA – ZEN – AGCCTAACAGGG – IABkFQ  AATGAGAGCCTCGTTCAAGC  GGCTTTTAGGTCACAGCACA  6FAM – AGAAGTTCTTAATTCCAAACCCTGA – BHQ1  ACGATGCTCATCACCTTTGC  TGCATGACCAGACCAGAGAT  6FAM – CGCATACCAAAGGGCACCCAATT – BHQ1 |

**Table S2.** Concentrations (copies/µl) of common (both males and females) and male specific allele for L68 and the common-to-male (C/M) ratio from mock sex ratio samples. STDV: standard deviation, RSD%: relative standard deviation.

|  |  | **L68** | | |
| --- | --- | --- | --- | --- |
| **Ratio** | **Series** | **Common** | **Male** | **Ratio C / M** |
| 1M | A | 497.2 | 62.7 | 7.9 |
|  | B | 558.8 | 177.1 | 3.16 |
|  | C | 652.3 | 80.3 | 8.1 |
| 3M:1F | A | 697.4 | 105.6 | 6.6 |
|  | B | 607.2 | 70.4 | 8.6 |
|  | C | 597.3 | 69.3 | 8.7 |
| 2M:1F | A | 682 | 74.8 | 9.2 |
|  | B | 673.2 | 96.8 | 7 |
|  | C | 606.1 | 71.5 | 8.5 |
| 1F:1M | A | 663.3 | 38.5 | 17.3 |
|  | B | 755.7 | 105.6 | 7.1 |
|  | C | 619.3 | 40.7 | 15 |
| 2M:2F | A | 522.5 | 44 | 11.8 |
|  | B | 645.7 | 56.1 | 11.6 |
|  | C | 617.1 | 46.2 | 13.3 |
| 2F:1M | A | 705.1 | 24.42 | 29 |
|  | B | 695.2 | 57.2 | 12.3 |
|  | C | 621.5 | 23.54 | 26 |
| 3F:1M | A | 617.1 | 18.59 | 33 |
|  | B | 696.3 | 52.8 | 13.1 |
|  | C | 766.7 | 18.48 | 42 |
| 1F | A | 672.1 | 0 | x |
|  | B | 650.1 | 0 | x |
|  | C | 513.7 | 0 | x |
|  | **Average** | 638.87 |  |  |
|  | **STDV** | 69.31 |  |  |
|  | **RSD %** | 10.85 |  |  |

**Table S3.** Concentrations (copies/µ) of common and male allele for L68, their common-to-male (C/M) ratio and concentrations (copies/µl) of mtDNA marker ND4 from eDNA samples. Reps denotes number of replicates.

|  |  | **ND4** | **L68** | | |
| --- | --- | --- | --- | --- | --- |
| **Ratio** | **Sample** | **mtDNA** | **Common** | **Male** | **Ratio C / M** |
| 1M | 1A1 | 874.50 | 153.58 | 10.47 | 14.67 |
|  | 1A2 | 193.60 | 7.02 | 0.17 | x |
|  | 1A3 | 212.30 | 54.02 | 19.63 | 2.75 |
| 2M:1F | 2A1 | 1180.30 | 287.79 | 72.36 | 3.98 |
|  | 2A2 | 515.90 | 25.12 | 1.58 | 15.90 |
|  | 2A3 | 2336.40 | 391.38 | 67.26 | 5.82 |
| 3M:1F | 3A1 | 4785.00 | 1428.48 | 260.45 | 5.48 |
|  | 3A2 | 1321.10 | 165.90 | 24.60 | 6.74 |
|  | 3A3 | 2289.10 | 237.58 | 37.15 | 6.40 |
| 1M:1F | 4A1 | 210.10 | 2.43 | 0.17 | x |
|  | 4A2 | 717.20 | 179.84 | 35.58 | 5.05 |
|  | 4A3 | 1289.20 | 127.91 | 26.47 | 4.83 |
| 1F | 5A1 | 533.50 | 8.02 | 0.00 | x |
|  | 5A2 | 104.50 | 3.98 | 0.00 | x |
|  | 5A3 | 356.40 | 21.80 | 0.00 | x |
| 1M:2F | 6A1 | 1403.60 | 48.90 | 1.06 | 46.11 |
|  | 6A2 | 1019.70 | 55.33 | 1.75 | 31.61 |
|  | 6A3 | 2816.00 | 146.15 | 8.57 | 17.06 |
| 1M:3F | 7A1 | 859.10 | 16.13 | 0.27 | x |
|  | 7A2 | 6182.00 | 2121.72 | 3.50 | 605.90 |
|  | 7A3 | 1646.70 | 148.00 | 16.27 | 9.10 |
| 2M:2F | 8A1 | 1602.70 | 274.34 | 18.45 | 14.87 |
|  | 8A2 | 407.00 | 75.63 | 15.29 | 4.95 |
|  | 8A3 | 1146.20 | 63.60 | 2.28 | 27.86 |
| Control | 9A1 | 0.00 | 0.00 | 0.00 | x |
|  | 9A2 | 0.00 | 0.00 | 0.00 | x |
|  | 9A3 | 0.00 | 0.00 | 0.00 | x |
|  | **ddPCR :** | 3 reps | 6 reps | | |

**Table S4.** Concentrations (copies/µl) of common and male nuclear markers and the ratio of common markers from DNA samples. STDV: standard deviation, RSD%: percentage of relative standard deviation.

|  | **Common** | | | **Male** | **Ratios** | |
| --- | --- | --- | --- | --- | --- | --- |
| **Sample** | **L68** | **DN59906** | **DN55850** | **L68** | **L68/ DN59906** | **L68/** **DN55850** |
| female | 1049.9 | 109.9 | 83.4 | 0.0 | 9.6 | 12.6 |
| female | 1429.6 | 123.7 | 91.0 | 0.0 | 11.6 | 15.7 |
| female | 1159.7 | 102.8 | 88.4 | 0.0 | 11.3 | 13.1 |
| male | 1090.2 | 77.1 | 81.5 | 340.2 | 14.1 | 13.4 |
| male | 930.4 | 55.6 | 41.9 | 222.3 | 16.7 | 22.2 |
| male | 948.2 | 49.7 | 71.2 | 127.7 | 19.1 | 13.3 |
| male | 703.2 | 43.6 | 54.2 | 224.4 | 16.1 | 13.0 |
|  |  |  |  | **Average** | 14.1 | 14.8 |
|  |  |  |  | **STDV** | 3.4 | 3.4 |
|  |  |  |  | **RSD%** | 24.4 | 23.3 |
