# Supplementary material for: Unlocking Demography: Developing an eDNA‐Based Toolkit to Measure Sex Ratios From Populations
Source: Mol Ecol Resour. 2025 Dec 18;26(1):e70089. doi: 10.1111/1755-0998.70089 (PMC12715386; doi:10.1111/1755-0998.70089)
Supplement: Supplementary file 1 — Data S1: men70089‐sup‐0001‐Supinfo.zip. [file MEN-26-e70089-s001.zip › men70089-sup-0001-FigureS1-S5.docx]

**Supplementary Figures**


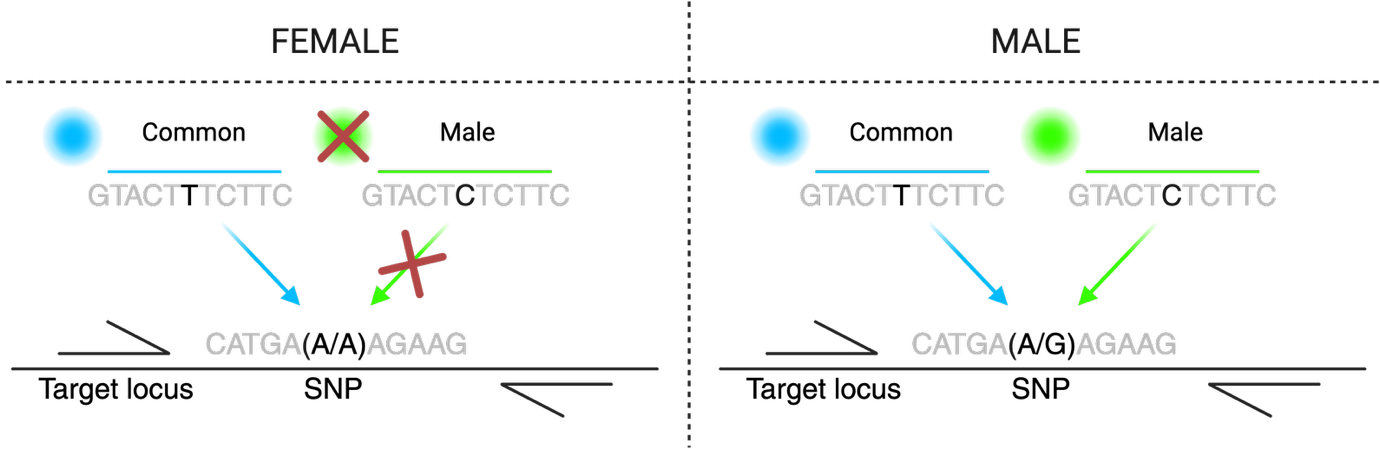


**Figure S1.** Representation of how our Rare Mutation Detection (RMD) ddPCR assay works; each duplex reaction contains two competitive fluorescent labeled probes targeting the two SNP alleles (common and male). For homozygous individuals (females) we only get fluorescent signal from the common allele probe (blue), whereas for heterozygous individuals (males) we get fluorescent signal from both common and male allele probes (blue and green). Created with [BioRender.com](https://www.biorender.com/)


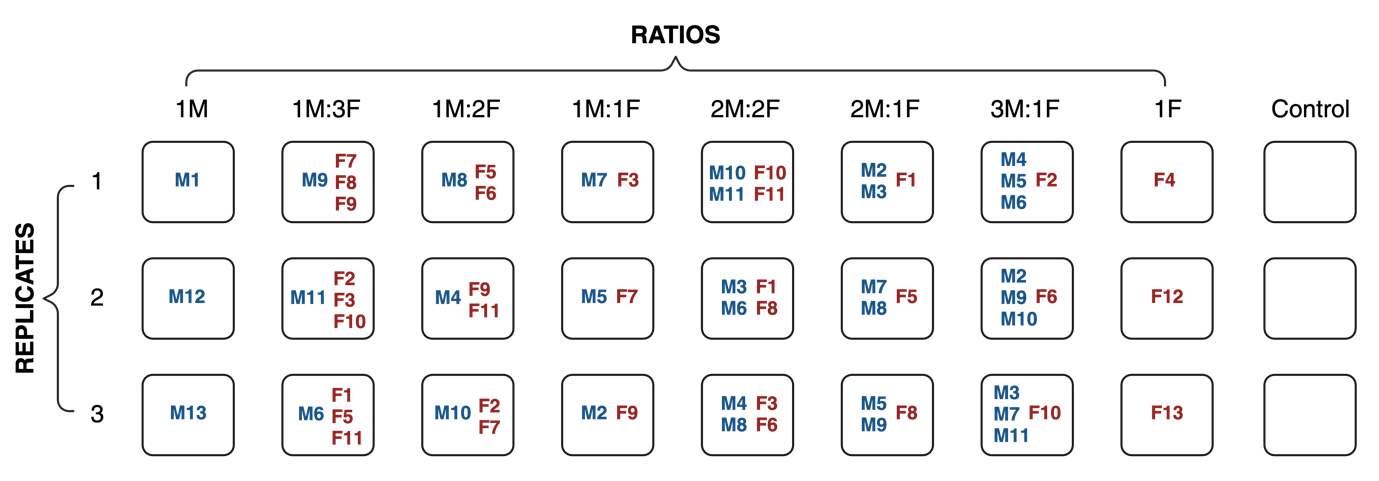


**Figure S2.** Representation of individuals (Triturus ivanbureschi) used in the eDNA sex ratio experiments. M denotes male and F female. Control treatment did not include any individuals. Each sample (square) was measured in six replicates. Created with [BioRender.com](https://www.biorender.com/)


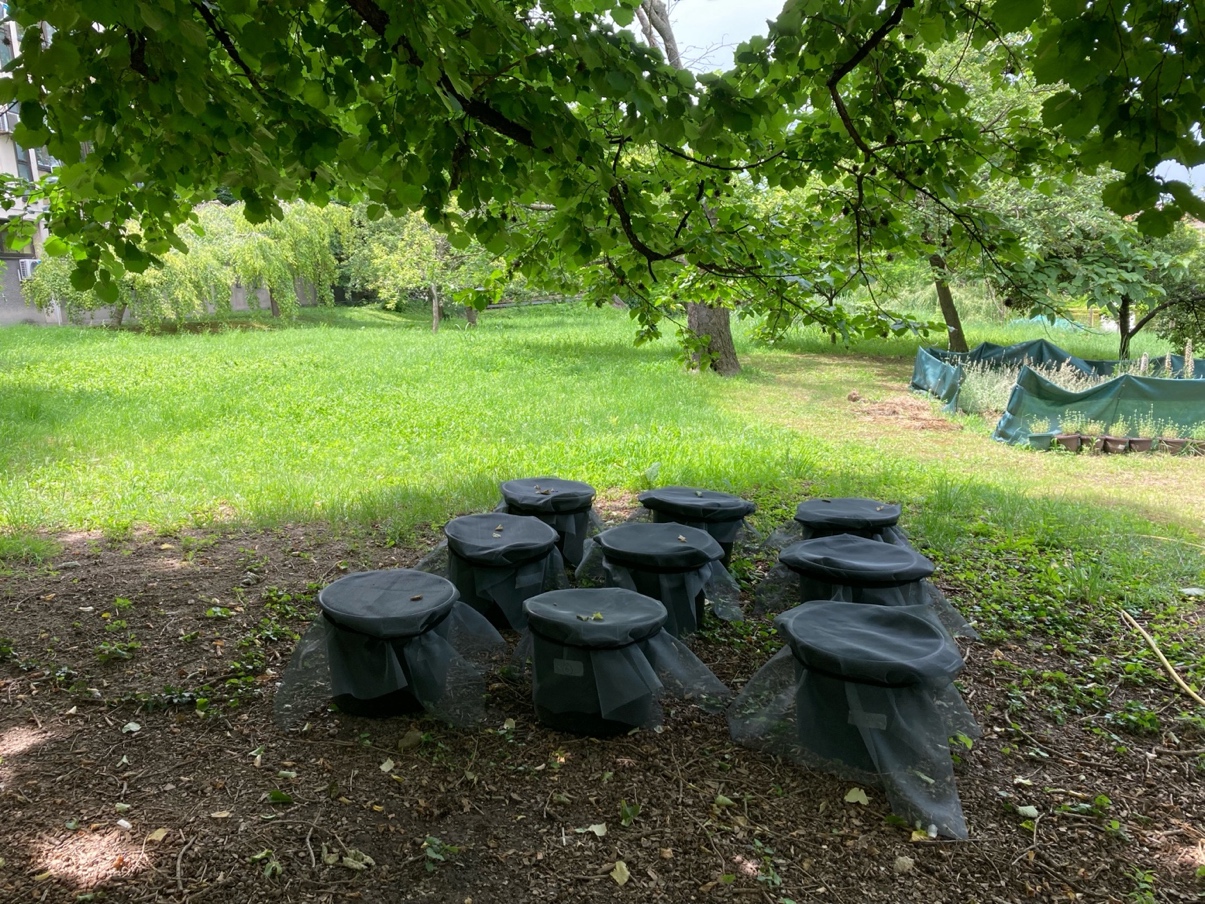


**Figure S3.** Set up of eDNA experiment at the Institute for Biological Research “Siniša Stanković” (University of Belgrade). Each replicate set up consisted of 9 containers (8 sex ratio treatments + 1 control; Figure S2). Containers were placed outside under a big tree providing shade.


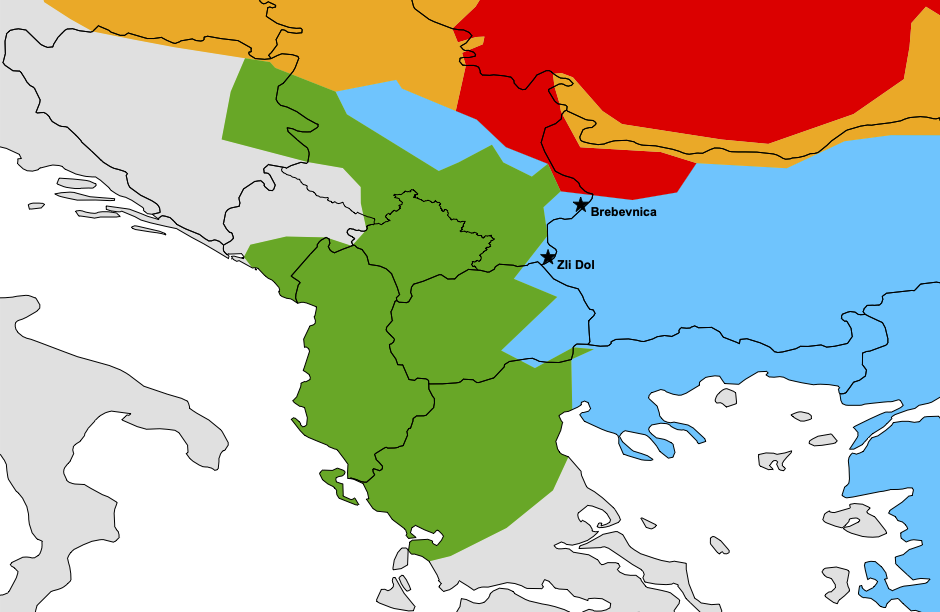


**Figure S4.** Map showing the distribution of *Triturus* species in the Balkans: *T. ivanbureschi* (blue), *T. macedonicus* (green), *T. cristatus* (red) and *T. dobrogicus* (orange). The stars represent the two locations in Serbia (close to Bulgarian borders) where individuals for this experiment originated in nature.


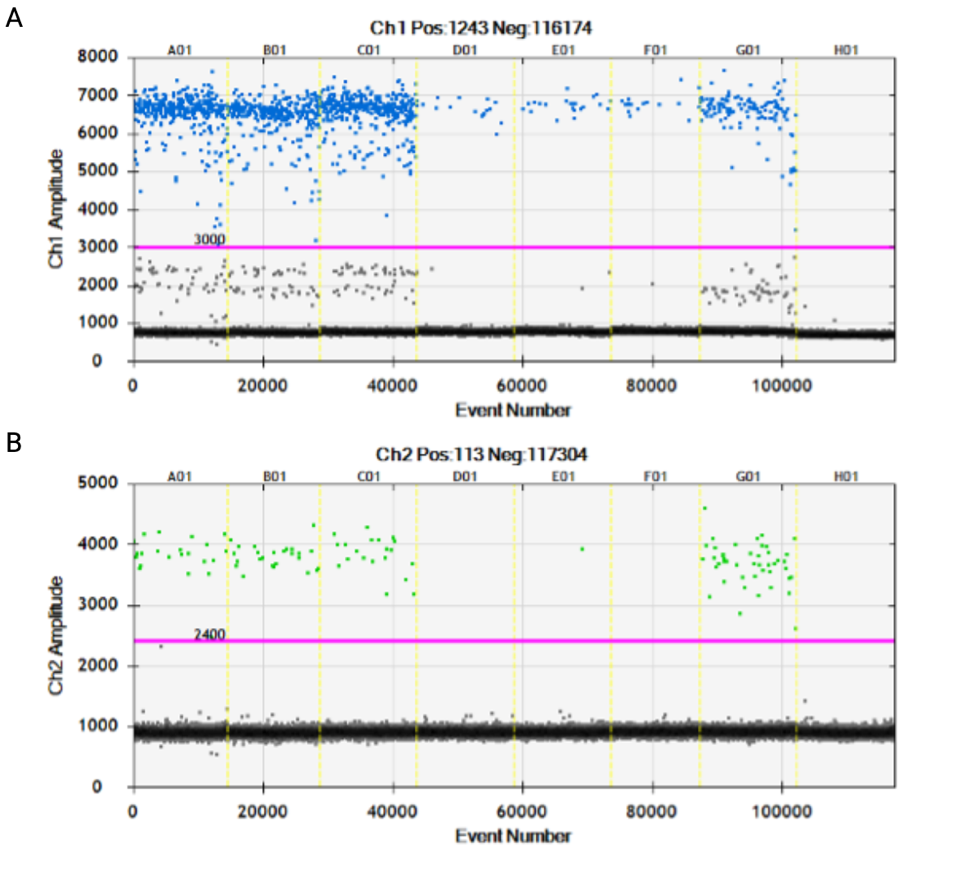


**Figure S5.** Representation of how fluorescence thresholds were manually set on QuantaSoft software (version 1.7; Bio-Rad Laboratories) for the common (A) and male (B) allele. Droplets above the threshold (pink line) are considered positive and droplets below the threshold as negative. Well H01 is a no-template control (NTC). The additional bands with low fluorescence signal below the threshold for the common allele are due to a combination of fluorescence bleed-through from the HEX channel (B) to the FAM channel (A) and non-specific binding to a different variant of the common allele sequence (Huggett, 2020; Whale et al., 2016).

Huggett, J. F. (2020). The digital MIQE guidelines update: minimum information for publication of quantitative digital PCR experiments for 2020. *Clinical chemistry*, *66*(8), 1012-1029.

Whale, A. S., Huggett, J. F., & Tzonev, S. (2016). Fundamentals of multiplexing with digital PCR. *Biomolecular detection and quantification*, *10*, 15-23.
